# An Innovative Multilevel Test for Hemoglobinopathies: TGA/Chemometrics Simultaneously Identifies and Classifies Sickle Cell Disease From Thalassemia

**DOI:** 10.3389/fmolb.2020.00141

**Published:** 2020-07-17

**Authors:** Roberta Risoluti, Patrizia Caprari, Giuseppina Gullifa, Sara Massimi, Laura Maffei, Francesco Sorrentino, Elena Carcassi, Stefano Materazzi

**Affiliations:** ^1^Department of Chemistry, Sapienza - University of Rome, Rome, Italy; ^2^National Centre for the Control and Evaluation of Medicines, Istituto Superiore di Sanità, Rome, Italy; ^3^Thalassemia Unit, S. Eugenio Hospital, Rome, Italy

**Keywords:** hemoglobinopathies, screening, thermogravimetric analysis, chemometrics, thalassemia, sickle cell disease

## Abstract

**Introduction:** Hemoglobinopathies are the most common genetic disorder wordlwide and because of migrations are become an emerging global health problem. Screening programmes for Sickle cell disease and Thalassemia have been implemented in some countries, but are not a common practice, due to a lack in the accuracy of the methods and to the costs of the analyses.

**Objectives:** The objective of this study was the application of the thermogravimetry coupled to chemometrics as new screening method to perform an early diagnosis of thalassemia and sickle cell disease.

**Methods:** Whole blood samples (30 μL) from sickle cell anemia and thalassemia patients were analyzed using the thermobalance TG7 and the resulting curves were compared with those of healthy individuals. A chemometric approach based on Principal Components Analysis (PCA) was exploited to enhance correlation between thermogravimetric profiles and a model of prediction by Partial Least Square Discriminant Analysis (PLS-DA) was developed and validated.

**Results:** The characteristic profile of the blood sample thermal decomposition and the first derivative of the TG curve showed that patients were clearly distinguished from healthy individuals as a result of different amounts of water and corpuscular fraction of blood. The chemometric approach based on PCA allowed a quick identification of differences between healthy subjects and patients and also between thalassemic and sickle cell anemia subjects. Chemometric tools (PLS-DA) were used to validate a model of prediction to process the thermogravimetric curves and to obtain in 1 h an accurate diagnosis. The TGA/Chemometric test permitted to perform first level test for hemoglobinopathies with the same accuracy of confirmatory analyses obtained by the molecular investigation.

**Conclusions:** A screening test based on the coupling of thermogravimetry and chemometrics was optimized for the differential diagnosis of hemoglobinopathies. The novel test is able to simultaneously perform a simple and fast diagnosis of sickle cell anemia or thalassemia, in a single analysis of few microliters of non-pretreated whole blood at low cost, and with high accuracy. Moreover this method results particularly suitable in pediatric patients as it requires small sample volumes and is able to characterize also transfused patients.

## Introduction

Hemoglobinopathies are the most common genetic disorder wordlwide, and include the Sickle Cell Disease (SCD) (Schnog et al., 2004; Rees et al., 2010; Kato et al., 2018), caused by structural changes in the globins chains of hemoglobin (Hb), and the thalassemia syndromes (Weatherall and Clegg, 2001a; Thein, 2013) that are disorders of globin chains expression (Schechter, 2008; Steinberg, 2008; Kohne, 2011).

SCD is a disorder of hemoglobin synthesis characterized by the production of an altered form of hemoglobin, hemoglobin S (HbS) for a mutation in the sixth codon of the β globin gene, which results in the substitution of glutamic acid for valine. Sickle hemoglobin, under hypoxic conditions, polymerizes leading to the sickling of the red blood cells. The most severe form is homozygous HbSS (Sickle cell anemia, SCA), but there are other compound heterozygous conditions such as HbS and β-thalassemia, or HbS and other Hb variants. Chronic anemia, hemolysis, and recurrent acute vaso-occlusive crises, characterized by pain and systemic inflammatory response, are the main clinical features (Azar and Wang, 2017; Ware et al., 2017).

Thalassemia syndromes are characterized by the absence or reduced β-globin chain synthesis, are heterogeneous at the molecular level with almost 300 point mutations and deletions classified as severe, mild and silent that can produce clinical and hematological phenotypes of variable severity ranging from the asymptomatic carrier to the severe transfusion-dependent type. Homozygosity or compound heterozygosity for β-thalassemia mutations cause a severe spectrum of anemias called thalassemia intermedia and thalassemia major (Cao and Galanello, 2010).

Hemoglobinopathies are particularly frequent in the countries where the malaria was endemic (Africa and Mediterranean region, Middle East, India, and South and East Asia) as a result of the heterozygote advantage against malaria. In the last decades, due to migrations, the hemoglobinopathies are become an emerging global health problem, and programs of screening for SCD and Thalassemia have been implemented in some countries (WHO report, 2005-2011; Weatherall, 2010; Williams and Weatherall, 2012).

The major limits to the expansion of these screening programs are the high cost and technical complexity of conventional diagnostics methods, specially for the developing countries where hemoglobinopathies are more frequent but poor facility for diagnosis, control and management of the subjects affected by hemoglobin disorders are available (Weatherall and Clegg, 2001b; Cao and Kan, 2012).

Laboratory diagnosis of hemoglobin disorders require a two levels diagnostic protocol (Clarke and Higgins, 2000; Cao et al., 2002; Greene et al., 2015; Sabath, 2017; Aiello et al., 2018). The first level screening tests include: (i) a complete blood count (CBC), since the hematological parameters mean corpuscolar volume (MCV), mean corpuscular hemoglobin (MCH), red blood cell counts (RBC), and red cell distribution width (RDW), are considered indicator in the haemoglobinopathies screening; (ii) the assessment of martial state to exclude the iron deficiency anemia; (iii) the study of hemoglobin fractions HbA_2_ and HbF by HPLC or electrophoresis to identify β-thalassemia phenotype, or abnormal peak for the presence of hemoglobin variants.

These methods permit the detection of the most common Hb variants (HbS, HbC, and HbE) that may result in a Hb disorder by interacting with β-thalassemia. Nevertheless, these methods do not detect the β-thalassemia in the newborn period, since HbA_2_ does not reach adult levels until about 6 months of age, and cannot be applied in transfused patients. A positive screening test requires to perform the second level tests to confirm the presence of hemoglobinopathy by molecular analysis of the globin genes (Mosca et al., 2009; Giardine et al., 2014; Barret et al., 2017). Therefore, the conventional screening methods for SCD and Thalassemia require cost, equipment and specialized personnel available only in few specialized clinical laboratories (Clarke and Higgins, 2000; Urrechaga et al., 2011).

Thermogravimetric techniques have been largely applied to complex matrix as analytical tool that does not require sample pretreatment nor clean-up (Toth et al., 2010; Fonseca et al., 2012; Materazzi et al., 2015, 2017a; Papadopoulos et al., 2016; Risoluti et al., 2016b, 2017; Bach and Chen, 2017; Catauro et al., 2018) and permits to rapidly obtain both qualitative and quantitative outcomes (Skreiberg et al., 2011; Materazzi et al., 2014a,b; Napoli et al., 2014; Shan-Yang et al., 2015; Marcilla et al., 2017). Recently, a novel approach based on the association of thermogravimetric analysis (TGA) and chemometrics has been proposed in different fields (Pappa et al., 2003; Khanmohammadi et al., 2012; Caramés-Alfaya et al., 2013; Chauhan et al., 2020), including the investigation of biological samples for clinical analysis (Risoluti et al., 2016a, 2018a; Materazzi et al., 2017a). Themogravimetry, in fact, with respect to clinical specimens, presents the advantage of processing samples without requiring any sample manipulation and the resulting curves reflect the characteristic composition of the sample in few microliters of sample. In addition, the coupling with chemometric tools permits to simultaneously consider the TG profile of blood samples and to compare in a click a patient of unknow diagnosis with the developed model of prediction (Risoluti et al., 2018c, 2019).

The objective of this study was the application of the thermogravimetry coupled to chemometrics, the TGA/Chemometric test, as new screening test to perform an early diagnosis of Thalassemia and SCD on few microliters of whole blood. This innovative screening test is cheap, specific, rapid, and applicable also in the early neonatal period, and in transfusion dependent people.

## Methods

### Enrollment of Patients

The study included a number of 235 subjects: among these, 120 healthy donors were characterized at the National Health Institute of Rome and 115 patients affected by hemoglobinopathies were followed for diagnosis, management and therapies at the Thalassemia Unit of S. Eugenio Hospital in Rome. The anemic patients included 65 subjects with Sickle Cell Anemia (SCA) diagnosis and 50 subjects affected by Thalassemias.

Blood specimens were collected in ethylene diamine tetracetic acid (EDTA) after informed consent of the patient (provided on request) and according to guidelines established by the Ethical Committee for human subject studies (Helsinki Declaration of 1975, revised in 2008).

### Hematological Characterization

The hematological characterization of all the collected samples from healthy subjects and patients was performed by evaluating the parameters RBC counts, Hb values, Hematocrit (Hct), MCV, MCH, and red cell distribution width (RDW) determined with an automated hematology analyzer ADVIA 120 (Siemens, USA). The comparison between the groups was performed by Student *t*-test for unpaired data.

### Thermogravimetric Analysis (TGA)

A Perkin Elmer TGA7 Thermobalance (Massachusetts, USA) was used to record the thermogravimetric curves. Non pre-treated whole blood (about 30 μl) was placed into the crucible where temperature was raised from 20 to 800°C, with a 10°C/min heating rate, as the best resolution rate. The atmosphere was air as carrier gas, at a flow rate of 100 ml/min. The Curie-point transitions of standard metals were involved to calibrate the instrumental response, as reported by the equipment recommendations. Reproducibility was checked by collecting three replicates for each sample. Derivative Thermogravimetric data (DTG) were also calculated to compare samples and represent the derivative of the function TG(T) with respect T.

### Chemometrics

Multivariate statistical analysis was performed by chemometrics with the aim of investigating correlations among samples and developing a model of prediction of the anemic status. Principal Component Analysis [PCA (Massart et al., 1998a; Ferreiro-González et al., 2018; Risoluti et al., 2018b)] was used as exploratory tool, while the classification model of prediction was pointed out by Partial Least Square Linear Discriminant Analysis [PLS-DA (Savitzky and Golay, 1964; Massart et al., 1998b; Barker and Rayens, 2003; Materazzi et al., 2017b,c)].

A chemometric study was planned with the aim of evaluating the most performing pretreatment to get the lowest root mean square errors (RMSE%) and the highest Specificity (Sp %) and Non Error Rate (NER %) (Murphy, 2012; Otto, 2017). The entire dataset of samples was divided into calibration set (75% of the dataset) and test set (25% of the dataset), while the prediction ability of the model were assessed by processing patients from an external dataset of measurements in order to ensure independency.

Diagnostics and acquisition of the thermogravimetric data were carried out by Pyris software (Thermo Fisher Scientific Inc., Waltham, MA, USA) as ASCII files, which were then imported into Unscrambler package to perform statistical analysis.

## Results

### Hematological Characterization

The hematological features were determined for all the collected samples from patients with confirmed diagnosis of sickle cell anemia (SCA) and thalassemia (T) and results were compared to those of healthy individuals.

The SCA group included patients with HbS homozygosity (HbSS), and with double heterozygosity (HbS/thalassemia, HbS/HbC, HbS/HbD). The thalassemia group included thalassemia major and intermedia patients, generally transfusion dependent, with different globin mutations β° and β^+^ in both homozygosity and heterozygosity.

Table 1 shows the hematological data expressed as mean ± standard deviation (SD). The group of SCA patients was characterized by a chronic hemolytic anemia with a decrease in the number of RBC and a corresponding decrease in the levels of Hb and Hct. MCV and MCH were significantly decreased, and the RDW values significantly higher, for the presence of hemolysis and reticulocytosis, in sickle cell patients than healthy donors (*p*-values: MCH = 0.01; all other parameters <0.0001). As expected, the comparison between thalassemic patients and healthy subjects showed significantly differences in all parameters (*p* < 0.0001) with decreased RBC, Hb, Hct, MVC, and MCH values and an higher RDW value.

**Table 1:** Hematological characterization of healthy individuals, sickle cell disease (SCA) and thalassemia (T) patients.

	**RBC (10^**6**^/mL)**	**Hb (g/dL)**	**Hct (%)**	**MCV (fL)**	**MCH (pg)**	**RDW (%)**
CTR	5.3 ± 0.4	14.8 ± 0.8	46.6 ± 2.8	87.4 ± 3.2	27.8 ± 1.4	13.2 ± 0.7
T	4.1 ± 1.1	10.0 ± 1.7	32.0 ± 6.1	79.9 ± 8.0	25.2 ± 3.5	18.3 ± 4.4
SCA	3.8 ± 0.7	9.9 ± 1.3	30.9 ± 3.8	81.8 ± 8.4	26.4 ± 3.3	20.0 ± 4.2
T vs. CTR^*^	3.3E-09	2.8E-26	1.3E-22	4.5E-07	3.9E-05	1.5E-10
SCA vs. CTR^*^	4.8E-26	2.1E-44	1.3E-47	7.1E-05	1.0E-02	1.1E-18
SCA vs. T^*^	1.1E-01	7.7E-01	2.2E-01	2.5E-01	7.5E-02	4.5E-02

* *p-value estimated to 95% of confidential level*.

Despite the investigation of the hematological parameters revealed that patients affected by hemoglobinopathies significantly differ to the population of healthy individuals a statistically difference between SCA and T subjects was observed only for RDW values (*P* < 0.05). Therefore, the hematological parameters alone did not differentiate patients with thalassemia from those with sickle cell anemia. This observation is in agreement with the heterogeneity and wide range of severity of both thalassemia and SCA patients, and in addition a number of HbS/Thal patients are present in SCA group.

### Thermogravimetric Analysis

**Figure 1:**
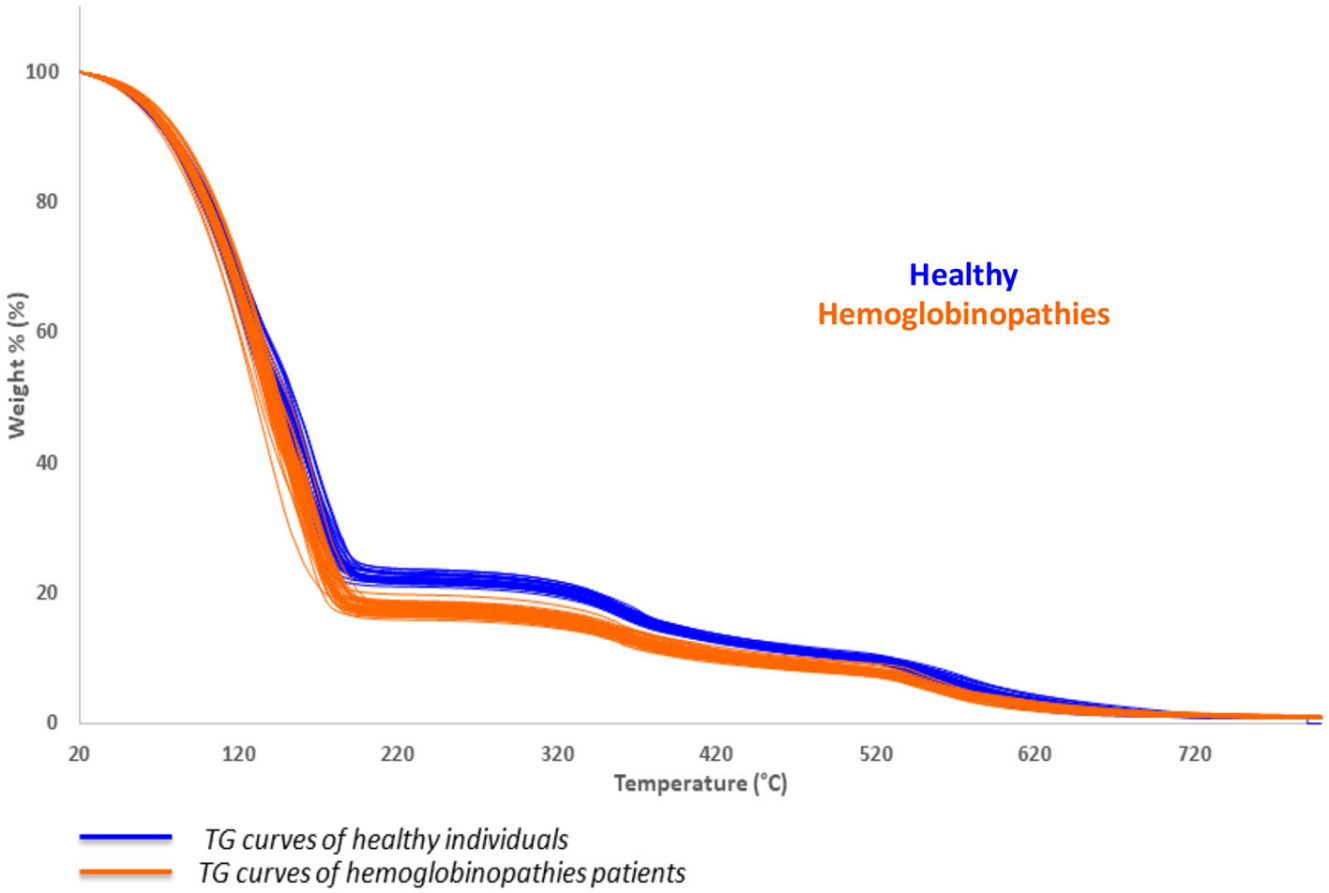
Collected thermogravimetric curves of healthy subjects (blue) and patients affected by hemoglobinopathies (orange).

Thermogravimetric analysis was performed on all the collected samples and the TG profile of blood samples was observed under controlled conditions of combustion. The characteristic thermal behavior of all patients affected by hemoglobinopathies (thalassemia and SCA patients) was estimated and compared to the thermal profile of healthy subjects, as reported in Figure 1.

A significant shift to lower temperature may be observed in the first thermally induced decomposition step of the patient with hemoglobinopathies (orange), with respect to healthy subjects (blue), as a result of the changing in the mass of the sample. The calculation of the first derivative curve of the TG profile, permitted to highlight all the decomposition steps of the samples and to calculate the percentage weight losses of each process: the first, corresponds to the release of the bulk water while the second to the bound water in the temperature range of about 50–150°C.

The total amount of water calculated for patients affected by hemoglobinopathies (sum of the first and second thermally induced processes) was found to be significantly higher than the percentage of the healthy subjects (*p*-values of 2.7E-22 and 3.7E-31 for thalassemia and SCA patients, respectively). On the contrary, the corpuscular fraction of blood related to the processes occurring at 350 and 530°C, was significantly lower in hemoglobinopathies than the healthy population (*p*-values of 7.6E-13 and 2.2E-11 for thalassemia patients and 1.7E-25 and 1.2E-15 for SCA patients). In addition, the decrease in the corpuscular fraction observed by TGA on hemoglobinopathies subjects (weight losses occurring at 350 and 530°C) is strictly associated to the anemia and hemolysis and is confirmed by the calculated hematological parameters reported in Table 1, where RBC, Hct, and Hb were found to be lower than the healthy donors.

The percentages of the bulk water and bound water were respectively higher and lower in SCA and thalassemic patients than the healthy subjects. As a consequence, the bulk/bound water ratio was found to be significant in differentiating SCA from healthy subjects (*p*-value of 4.7 E-03) and thalassemic from healthy individuals (*p*-value of 1.3 E-07). Interestingly, as suggested by the *p*-values reported in Table 2 where SCA and thalassemic groups are compared, both the two groups showed a similar distribution of the total amount of water (water content) and the corpuscular fraction (2nd and 3rd weight losses) as no statistically differences were observed in the corresponding TG parameters. On the contrary, the distribution of the bulk water and bound water was found to be significantly different as well as the bulk bound water ratio.

**Table 2:** Calculated weight losses (%) of each thermally induced decomposition process.

	**Water Content (%)**	**Bulk Water (%)**	**Bound Water (%)**	**Bulk/Bound Water ratio**	**2nd Weight Loss (%)**	**3rd Weight Loss (%)**
CTR	78.1 ± 0.9	46.8 ± 4.0	30.9 ± 4.8	1.6 ± 0.5	11.5 ± 0.7	9.1 ± 0.5
T	82.1 ± 1.7	62.8 ± 8.8	19.3 ± 8.1	4.3 ± 3.0	9.6 ± 1.1	7.8 ± 0.9
SCA	81.6 ± 1.3	55.7 ± 8.6	26.0 ± 8.3	2.8 ± 2.7	9.4 ± 0.9	7.8 ± 0.9
T vs. CTR^*^	2.7E-22	1.5E-16	2.6E-11	1.3E-07	7.6E-13	2.2E-11
SCA vs. CTR^*^	3.7E-31	4.2E-09	5.9E-04	4.7E-03	1.7E-25	1.2E-15
SCA vs. T^*^	8.2E-02	7.3E-05	8.7E-05	7.5E-03	2.5E-01	9.8E-01

* *p-value estimated to 95% of confidential level*.

These preliminary results suggested that the themoanalytical investigation of the blood may be more effective in discriminating SCD from thalassemia disease than the hematological features.

### Chemometric Analysis

**Figure 2:**
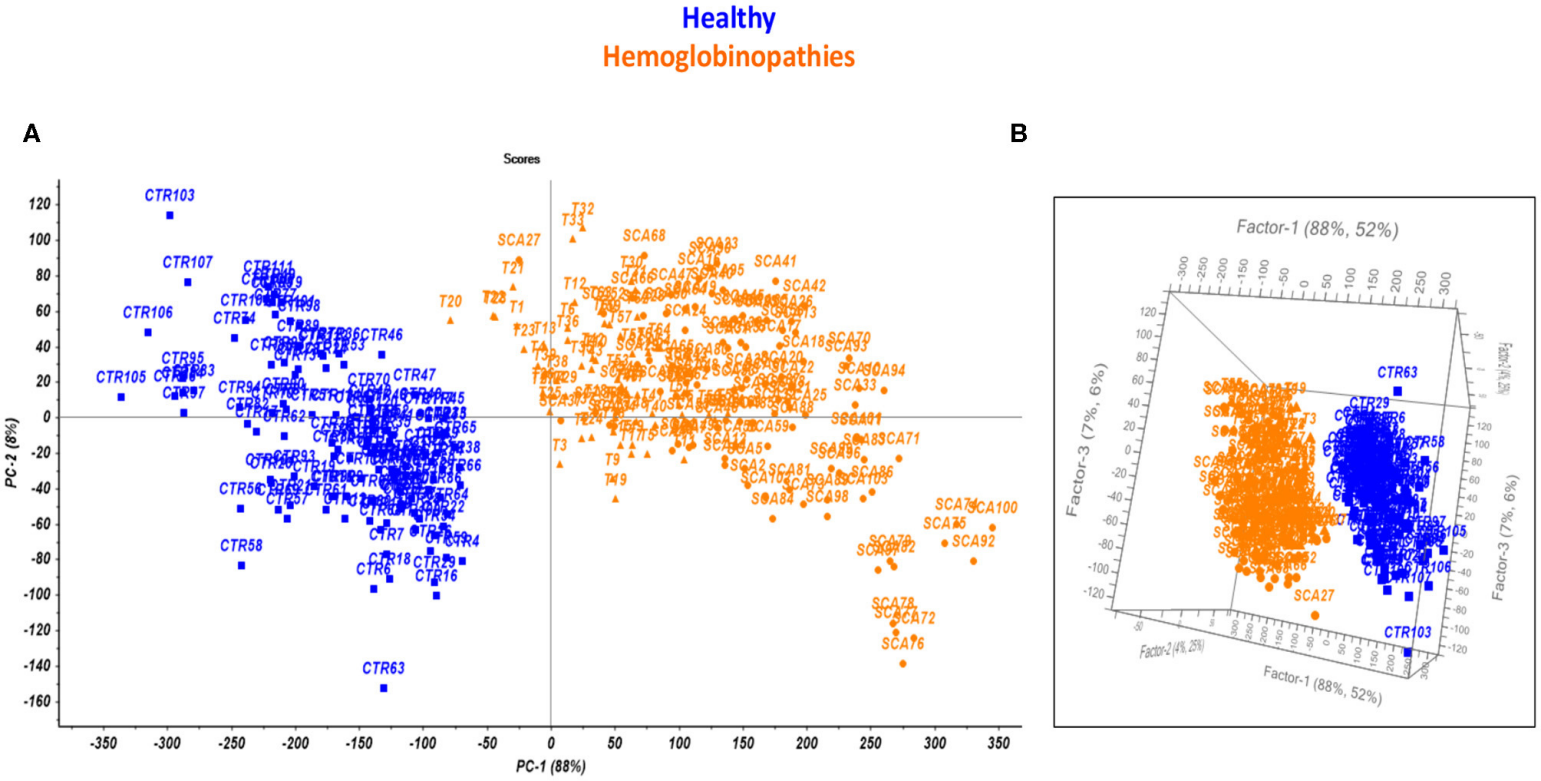
Scores plot performed by PCA **(A)** and PLS-DA model **(B)** on the TG curves for healthy subjects (blue) and patients affected by hemoglobinopathies (orange).

Statistical analysis was performed on the collected thermogravimetric curves, by principal component analysis (PCA), with the aim of highlighting correlations among measurements. The resulting scores plot, obtained after mean centering correction of data, is reported in Figure 2A. The mean centering data processing is performed by calculating the average vector of the curves of all the rows and subtracting it point by point from each vector in the dataset. The scores plot reported in Figure 2A, clearly shows two separated groups related to the healthy subjects (blue squares) and the patients affected by hemoglobinopathies (orange triangles). The two groups of subjects may be differentiated according to the first principal component that explains the maximum of the variance in the data set (88%). Therefore, the explorative multiparametric analysis of the TG curves performed by PCA (overall explained variance on of 96% using two PCs), permitted to put in evidence differences between healthy and subjects with hemoglobinopathies, as a function of the presence or the absence of the anemia.

A model of prediction was developed by Partial Least Square Discriminant Analysis (PLS-DA) in order to instruct a platform to rapidly identify the anemic status. To this aim, the 75 and 25% of the entire set of samples, were used as calibration and evaluation set, respectively. In addition, with the aim of improving accuracy and sensitivity of the method, different chemometric pre-treatments were investigated. Among these, Mean Centering (MC), Standard Normal Variate (SNV), and a combination of the first derivative of the TG curve and MC or SNV (Murphy, 2012; Otto, 2017).

The figures of merit of the model were calculated to evaluate the prediction ability of the novel test, including the Non Error Rate (NER) expressed as the percentage of samples correctly predicted by the model, the specificity (Sp %) expressed as the percentage of samples predicted as belonging to the correct class and the Root Mean Square Error (RMSE %). As a consequence, the most performing pre-treatment of the TG curves was selected as the one providing the highest values of NER % and Sp % and the lowest values of RMSE % in calibration and validation. The optimized model for the identification of the presence of anemia provided for an accuracy and a specificity of about 100% and the RMSE was 0.2%. for both controls and hemoglobinopathies. The resulting 3D plot of the model is reported in Figure 2B.

### Development of the Multilevel Test

**Figure 3:**
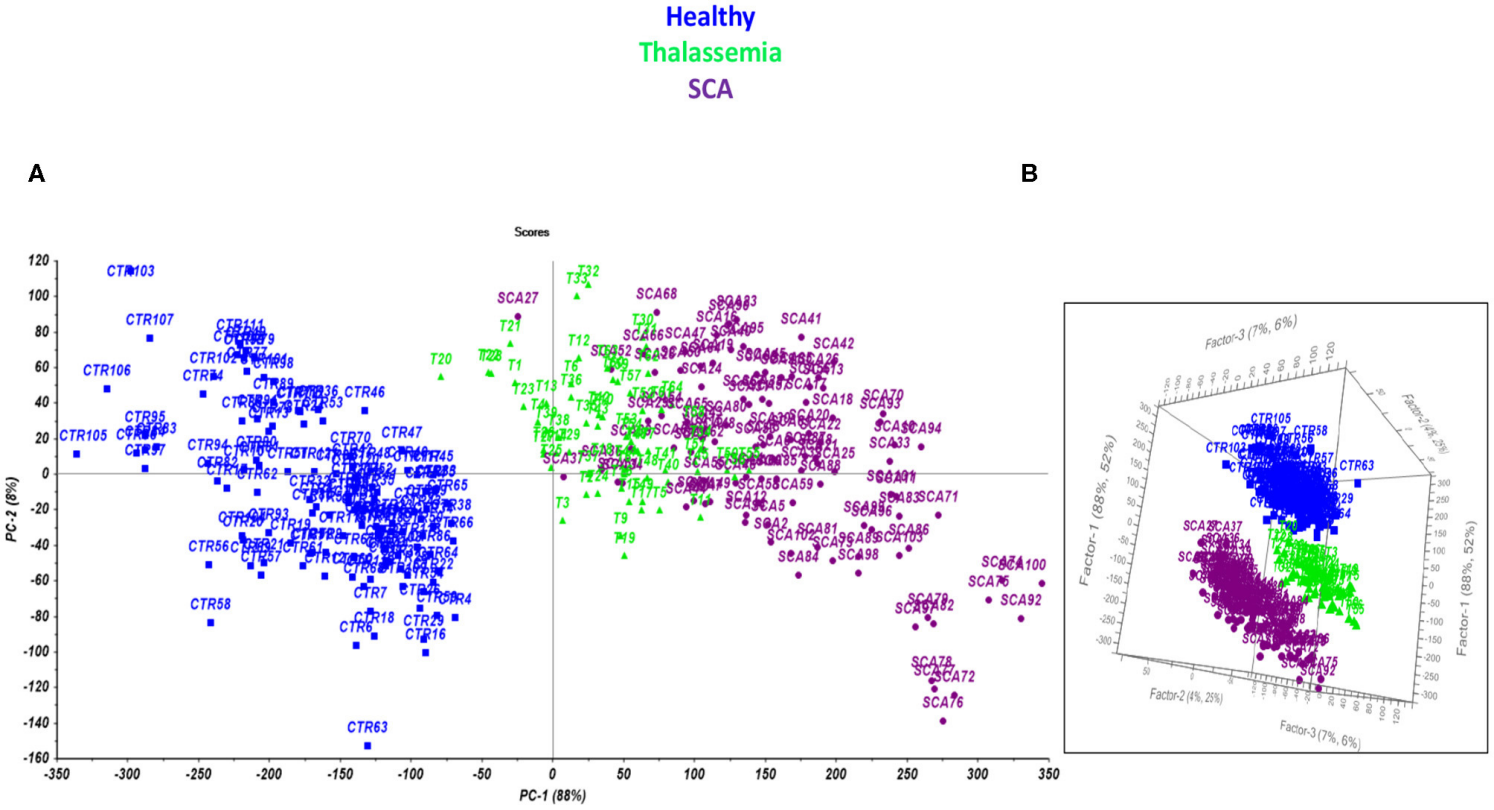
Scores plot performed by PCA **(A)** and PLS-DA model **(B)** on the TG curves for healthy subjects (blue), and patients affected by thalassemia (light green) and SCA (purple).

The second stage of the work focused on the optimization of a comprehensive model of prediction able to simultaneously identify and classify the presence of anemia, discriminating SCD from thalassemia. Preliminarily, a Principal Component Analysis was performed to evaluate the most performing conditions of separation of the samples (Figure 3A) and at last a model of prediction by PLS-DA was developed for the final multilevel test (Figure 3B).

The model permits to separate samples according to the different class they belong to (Healthy, SCA or thalassemic) and may represent a novel way to perform the screening of hemoglobinopathies with the accuracy (100% of NER for all the classes) and the specificity (100% of Sp for all the classes) of the second level test since all the subjects were correctly differentiated according to PC 1. The analysis of the factor loadings provided the clarification of the variables affecting most the distribution of samples, concluding that the range of temperature 85–190°C associated to the bulk and bound water release is responsible for SCA and thalassemia discrimination. In fact, the prediction ability of the model was estimated by the calculation of the figures of merit reported in Tables 3–5, showing that the involvement of the first derivative of the TG curves prior to mean centering pretreatment, permitted to obtain the suitable results in calibration, cross-validation and prediction of external set of samples, providing the lowest error of prediction (RMSE %) of about 0.18, 0.20, and 0.10% for healthy, talassemic and SCA subjects, respectively.

**Table 3:** Calculated Non Error Rate (NER %) of the model in calibration, validation and prediction, obtained using different chemometric pre-treatments.

	**Patient**	**Mean centering**	**SNV**	**1st derivative + mean centering**	**1st derivative + SNV**
Calibration	CTR	100.0	100.0	100.0	99.1
	T	100.0	98.4	100.0	95.3
	SCA	100.0	100.0	100.0	100.0
Validation	CTR	100.0	100.0	100.0	93.9
	T	96.9	92.2	95.3	85.9
	SCA	100.0	100.0	100.0	100.0
Prediction	CTR	100.0	100.0	100.0	100.0
	T	100.0	100.0	100.0	100.0
	SCA	100.0	100.0	100.0	100.0

**Table 4:** Calculated specificity (Sp %) of the model in calibration, validation and prediction, obtained using different chemometric pre-treatments.

	**Patient**	**Mean centering**	**SNV**	**1st derivative + mean centering**	**1st derivative + SNV**
Calibration	CTR	98.3	100.0	100.0	98.3
	T	100.0	95.1	98.3	93.3
	SCA	100.0	100.0	100.0	100.0
Validation	CTR	100.0	100.0	100.0	88.6
	T	93.4	85.2	91.8	83.0
	SCA	100.0	100.0	100.0	100.0
Prediction	CTR	100.0	100.0	100.0	100.0
	T	100.0	100.0	100.0	100.0
	SCA	100.0	100.0	100.0	100.0

**Table 5:** Calculated Root Mean Square Error (RMSE %) of the model in calibration, validation and prediction, obtained using different chemometric pre-treatments.

	**Patient**	**Mean centering**	**SNV**	**1st derivative + mean centering**	**1st derivative + SNV**
Calibration	CTR	0.19	0.20	0.18	0.18
	T	0.25	0.23	0.21	0.22
	SCA	0.12	0.10	0.11	0.12
Validation	CTR	0.20	0.20	0.20	0.23
	T	0.25	0.24	0.24	0.28
	SCA	0.13	0.12	0.12	0.12
Prediction	CTR	0.22	0.24	0.18	0.21
	T	0.18	0.18	0.20	0.22
	SCA	0.15	0.16	0.10	0.10

### Diagnosis of Difficult Cases of Anemia

The novel test was used to investigate some cases of hemolytic anemias of difficult diagnosis such as in neonatal period and in transfused patients. We have analyzed seven subjects, characterized by hemolytic anemia, negative Coombs tests, and increased LDH value that suggested the presence of an erythrocyte congenital defect as cause of the hemolytic anemia. The TGA/Chemometrics test showed the presence of two cases of SCA and five patients with thalassemia (two of them in the newborn period). The 2nd level tests by molecular analysis of the globin genes confirmed the presence of the globin genes mutations. All the analyzed patients were found to be correctly predicted by this screening test that permitted a differential diagnosis of two hemoglobinopathies. The application of this new screening method will allow to address patients to the 2nd level confirmatory analysis reducing time and costs for the diagnosis.

## Discussion and Conclusions

The disorders of hemoglobin, such as thalassemia and SCD, are today a real global healthy problem. A rapid and correct diagnosis becomes extremely relevant because allows an early preventive care and avoids consequences for affected children mainly in low and middle-income countries. A number of procedures have been proposed for screening hemoglobinopathies based on complete blood counts followed by the analysis of hemoglobin fractions, differential erythrocyte density, differential wicking of Hb S and Hb A through filter paper, a polyclonal antibody-based capture immunoassay, and next-generation sequencing (Korf and Rehm, 2013; McGann et al., 2016; Piety et al., 2016; Barret et al., 2017). Despite these methods contribute significantly in improving the first level test, they lack in 100% accuracy and the cost is prohibitive in low-resource countries.

This study introduces an innovative method to perform the screening of hereditary hemoglobin defects, characterized by severe hemolytic anemia with different severe clinical manifestations. Systematic screening programmes for hemoglobinopaties are not a common practice, due to a lack in the accuracy of the methods and to the costs of the analyses. As a consequence, the diagnosis is usually made when a severe complication occurs, particularly in developing countries. The novelty of this study consists of the accuracy and the simplicity of the test that permits to rapidly perform a differential diagnosis of sickle cell anemia and thalassemia. The study demonstrated that the themoanalytical investigation of the blood was more effective in discriminating SCA from thalassemia disease than the hematological features. The application of a model of prediction by PLS-DA was developed for the final multilevel test and this model permits to separate samples according to the different class they belong to (Healthy, SCA or thalassemic) and may represent a novel way to perform the screening of hemoglobinopathies with the accuracy and the specificity of the second level test since all the subjects were correctly differentiated.

In conclusion the application of TGA/chemometric analysis has proved to be a particularly useful diagnostic tool for the screening of the hemoglobin defects, in a short time and at low cost, also in case of congenital hemolytic anemia of difficult diagnosis. This method results particularly suitable in pediatric patients as it requires small sample volumes and is able to characterize patients subjected to transfusion.

## Data Availability Statement

The raw data supporting the conclusions of this article will be made available by the authors, without undue reservation.

## Ethics Statement

The studies involving human participants were reviewed and approved by Comitato Etico Roma 2 of the S. Eugenio Hospital, Rome. Written informed consent for participating in the study and publishing clinical data in an anonymized manner are collected and copies of the informed consent are available on request. In the case of child, written informed consent for participating in the study and publishing clinical data in an anonymized manner was obtained from the participant's legal guardian/next of kin.

## Author Contributions

RR, SMat, and PC conceived the study and wrote the manuscript. FS and LM enrolled the patients and performed the clinical and laboratory evaluation. GG, SMas, and EC performed analysis. All authors performed the data analysis, contributed to the article, and approved the submitted version.

## Conflict of Interest

The authors declare that the research was conducted in the absence of any commercial or financial relationships that could be construed as a potential conflict of interest.
